# Towards understanding *Cameraria ohridella* (Lepidoptera: Gracillariidae) development: effects of microhabitat variability in naturally growing horse-chestnut tree canopy

**DOI:** 10.1007/s00484-021-02119-8

**Published:** 2021-04-21

**Authors:** Piotr Łaszczyca, Mirosław Nakonieczny, Andrzej Kędziorski, Agnieszka Babczyńska, Marta Wiesner

**Affiliations:** 1 Faculty of Natural Sciences, Institute of Biology, Biotechnology and Environmental Protection, Animal Physiology and Ecotoxicology Team, University of Silesia in Katowice, PL 40-007 Katowice, Bankowa 9 Poland; 2 Główny Instytut Górnictwa (GIG), 40-166 Katowice, plac Gwarków 1 Poland

**Keywords:** Degree-days, Horse-chestnut leaf-miner, Image analysis, Microhabitat, Mine size, Model of development

## Abstract

Dwelling intensity of horse-chestnut miner (*Cameraria ohridella*) larvae in various leaves insolation and temperature was measured to determine whether this pest’s development follows a predictable pattern or depends more on local microenvironment conditions. Mines growing on leaves of mature host plants (*Aesculus hippocastanum* L.) in their natural conditions were photographed for two consecutive generations of the pest and in two separated vegetation periods. Apart from meteorological data obtained from the nearest station, the temperature of intact and mined parts of sun-exposed and shaded leaf blades was measured at various daytimes throughout the experiment. Obtained sets of digital data were analysed and combined to model mine area growth as a function of degree-days sum by adopting of Verhulst logistic equation. We showed the predictive potential of our model based on experimental data, and it may be useful in the scheduling of pest control measures in natural conditions. Our analyses also revealed that despite significant differences in microenvironment conditions depending on mines’ insolation, the horse-chestnut miner larvae could partially compensate for them and complete their development at similar endpoints expressed as the cumulative sum of degree-days. We conclude that computer-aided analysis of photographic documentation of leaf-miner larval growth followed by mathematical modelling offers a noninvasive, reliable, and inexpensive alternative for monitoring local leaf-miners populations.

## Introduction

The progressive invasion of horse-chestnut leaf-miner *Cameraria ohridella* Deschka and Dimic 1986 (Lepidoptera, Gracillariidae) in Europe since 1990s has stimulated much research on the pest’s biology, impact, and management (Tomiczek and Krehan 1998; Akimov et al. 2003; Gilbert et al. 2005; Kenis et al. 2005; Girardoz et al. 2007; Syeryebryennikov et al. 2008; Stygar et al. 2013; Pocock and Evans 2014). Across its present range that spans from Greece and Balkan countries (*C. cameraria* refugium until the end of the 1980s) to Scandinavia and England (invaded in 2000–2005), the pest has 2–4 partially overlapping generations during the vegetation period (Dimic et al. 2000; Buszko 2006; Pocock and Evans 2014). The rate of larval development and moulting results from the sum of effective temperatures above the lower threshold that is usually described as the sum of degree-days or growth degree-days (Dimic et al. 2000; Dautbašić 2006; Meshkova and Mikulina 2013). Recent molecular studies revealed small genetic variability among invasive populations compared with native horse-chestnut leaf-miner populations inhabiting the Balkans, considered the most likely area of the pest’s origin in Europe (Valade et al. 2009; Lees et al. 2011). Thus, any differences in the pest development on invaded areas can be merely ascribed to local variability of biotope conditions.

Although calculations of growth degree-days have been common practice for numerous pest species, predictions of leaf-miners development based on air temperature might be less accurate due to variation of local thermal conditions among particular mines depending on their exposure to sunlight (Pincebourde and Casas 2006a, b; Pincebourde and Woods 2012). ‘Thermal variation’ within local microenvironment affects performance (e.g. rate of development or activity) in numerous ectotherms, and leaf-dwellers are no exception here (Angilletta Jr. et al. 2002; Niehaus et al. 2012; Pincebourde and Woods 2012). Moreover, sun-exposed leaves have thicker mesophyll layers, hence greater biomass per unit area, than leaves growing in the shade; furthermore, they contain more secondary metabolites (Adams and Langton 2005; Fritz et al. 2018; Yang et al. 2018).

Larval development of *C. ohridella* depends on light conditions and position within the tree canopy (Birner and Bohlander 2004; Horváth and Benedek 2009). Recently, Jagiełło et al. (2019) in a glasshouse experiment demonstrated more extensive mine area in low light than in high light but the same mass eaten by the larvae due to the thicker leaf blade in high light conditions. However, a glasshouse experiment cannot mimic the complexity of microenvironment conditions that affect leaf-dwellers in their natural habitat. Moreover, it has been shown that senescence of autumn leaves increased *C. ohridella* larvae mortality (Samek 2003; Girardoz et al. 2007). Thus, the question remains whether the completion of larval development in sun-exposed vs shaded leaves depends more on the leaf ‘quality’ or amount of heat (sum of effective temperatures in microenvironment of particular larvae) in their natural conditions and whether it follows a predictable pattern.

We combined quantitative data of individual larvae mining of two consecutive generations with meteorological data to create a mathematical model of *C. ohridella* development in the growing season. Then we critically tested the model’s accuracy in predicting the pest phenology and its utility for timing control measures, e.g. applying pheromonal traps for moths.

## Material and methods

### Experimental setup and data collection of mine-dwelling activity

Observations were carried out in 2009 (5th May–23rd August) and 2013 (6th June–13th September) on mines of *C. ohridella* on *Aesculus hippocastanum* L*.* specimens, about 20 years old, growing in a large urban park (50.2912°N, 18.9774°E, Chorzów, Poland). Randomly selected young leaves, accessible from the ground, with different numbers of mines (in an initial stadium), were tape-marked on their petioles, numbered and classified into two groups, depending on their position within the canopy: *sun-exposed (Sun)* located on southern and superficial part of the canopy and not obscured by other leaves and *shaded (Shade)*—on the northern part of the foliage and void of direct sun rays. Other trees did not shade the selected *A. hippocastanum* canopies.

**Fig. 1:**
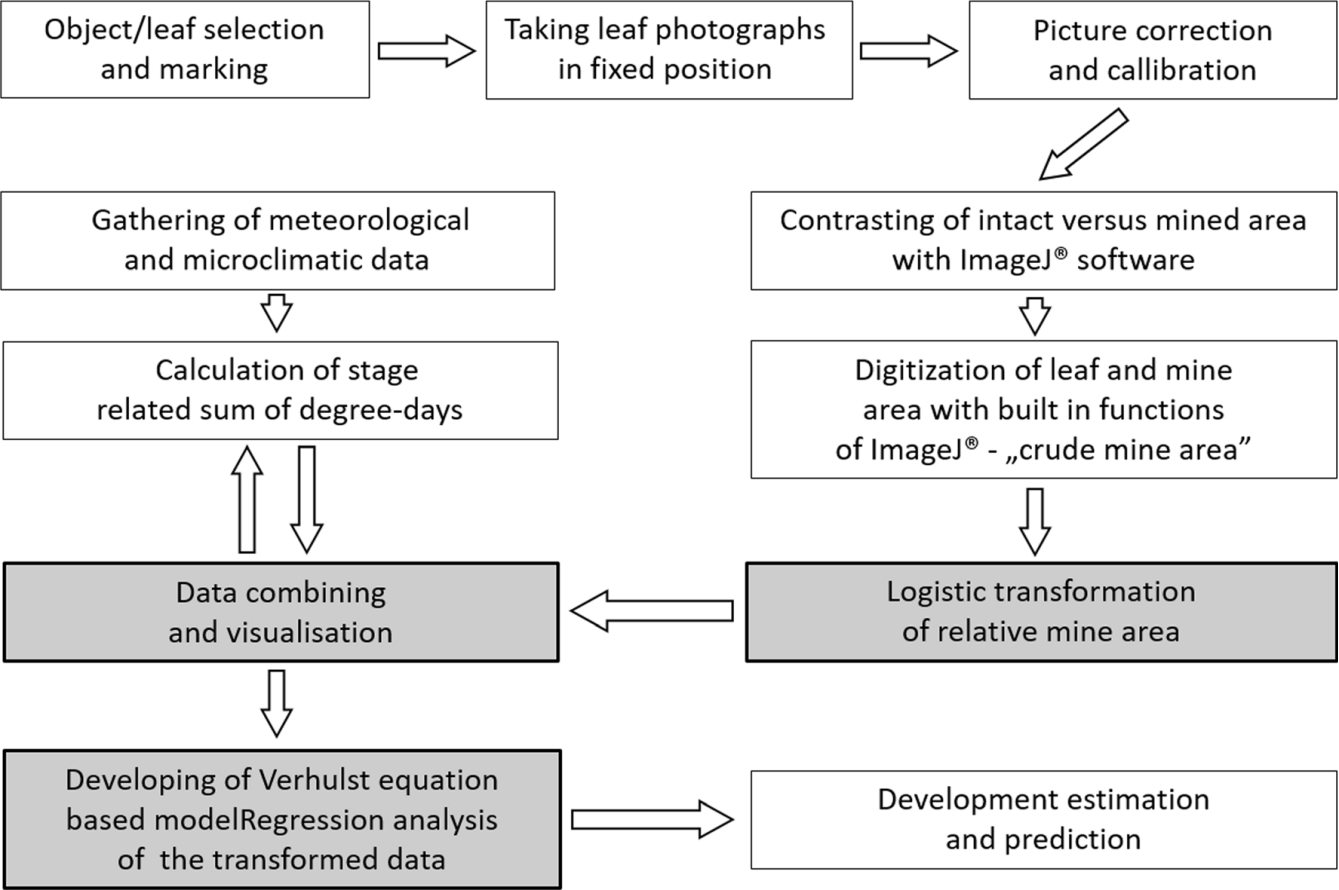
Subsequent steps of degree-day based model elaboration of leaf-miner *Cameraria ohridella* (Lepidoptera, Gracillariidae) infestation on host leaves

In 2009, we photographed 14 Sun and 8 Shade leaves 13 times each during the first generation (final moult on 23rd July) and 8 Sun and 8 Shade leaves 9 times during the second generation. Only partial examination of the second generation was possible due to accidental damage of our test foliage by the park service. We documented mine development with 1402 and 716 photographs of the leaves for the first and second generations (see below). In 2013, we photographed 10 Sun and 9 Shade leaves 8 times each during the first generation (6th June–17th July) and 9 Sun and 9 Shade leaves 8 times each during the second generation (26th July–28th August), so the mine’s development was documented with 1540 photographs altogether (Fig. 1). The caterpillars of all inspected mines pupated and finally emerged as mature moths. Thus we concluded that the larvae were not parasitised.

Additionally, mean fresh and dry mass per surface area was calculated for randomly selected 8–9 leaflets for each lighting condition at a particular date, with 52 samples altogether.

### Data elaboration and model construction

Images of mined leaves were captured with Olympus FE-20 camera (min. resolution 4600 × 3450 px, c.a. 4 MB per image) against a graph paper. Images were adjusted with ImageJ 1.41 software (http://imagej.nih.gov/ij/) and GIMP or CorelDraw® to correct the accidental distortion of the leaf plane. The adjustment included image scaling and filtering with built-in Object-Based Image Analysis modules of ImageJ® (for better contrast between mined and intact parts of the leaf and setting threshold values of the measured areas), followed by ‘automatic’ area measurements (in mm^2^). Data transformation to relative values eliminated problems with image distortion and calibration. The mines were measured either on randomly selected leaflet(s) of the leaf (in 2009) or all leaflets of the chosen leaves (in 2013).

The ImageJ® data for each experimental group (including the year of study, the pest generation, and the leaf ‘group’) were elaborated statistically for comparison and developmental model construction. Relative mine surface area (rA) in the period of generation growth was calculated, taking the maximum value of average mine surface for that generation as the basis for estimation. In the next step, rA (Sun/Shade) was plotted as a function of time (days) or sum of degree-days (see below). Verhulst logistic equation was subsequently applied to the data, and parameters of the equation were calculated with the appropriate regression model. The final linear regression model was built using MS Excel® formulas, based on Verhulst logistic equation (Eqs. (1) and (2)) after logit data transformation of average mine surfaces (Tsoularis 2001; Tsoularis and Wallace 2002; Vandermeer 2010):

1 $$ \frac{dN}{dt} = rN\cdotp \frac{K-N}{K} $$

2 $$ {N}_t=\frac{K}{1+{e}^{a- rt}} $$

where *N* is the variable describing temporary population growth (here = mine area), *K* is the corresponding maximal value (here = maximal mine area measured for given generation), *r* is the rate of growth/coefficient/, *t* is the independent variable (here = time or sum of effective temperatures), *a* is the constant of integration (from regression equation), and *e* is the base of natural logarithm.

Logit linear transformation was based on the following formulas (Eqs. (3) and (4)) (McDonald 2014):

3 $$ Logit\left({N}_t\right)=\frac{\mathit{\ln}{N}_t}{1-{N}_t} $$

4 $$ Logit\left({N}_t\right)=r\cdotp t-a $$

Parameters of the linear equation (slope, intercept, correlation coefficient, and error of estimation) were obtained by LSD methods and MS Excel® formulas.

### Collection of microclimatic data

The Central Mining Institute’s meteorological station in Katowice (50.2709°N, 19.0259°E—about 6 km away from the experimental stand) provided data including minimum and maximum daily temperature and air humidity, throughout the whole observation periods in 2009 and 2013.

Temperature data were used to calculate the sum of effective temperatures (day-degree sum). We chose 0 °C as the basis for calculations instead of about 8–10 °C assumed by some authors as low developmental threshold for *C. ohridella* and other Lepidoptera (Doganlar 2008; Nietschke et al. 2007; Meshkova and Mikulina 2013; Jarošík et al. 2015). Such an approach avoids the possible error of overestimating the actual developmental threshold that may be lower in the leaf-miner populations invading Europe in northward direction (see ‘Discussion’).

Estimated surfaces of the photographed mines were plotted against time from the onset of observations, or sum of effective temperatures.

Microclimatic data were obtained for the leaves of horse-chestnut trees growing in the experimental stand, Katowice Forest Park (50.2331°N, 19.0204°E), Kosciuszko Park (50.239881°N, 19.003296°E), and in the small town garden (50.2590°N, 19.0287°E). The canopy’s air temperature was measured about 2–5 cm over the leaf surface with a non-contact digital infrared sensor with ± 0.3 °C accuracies (model ST8806H, Standard Instruments Co., Ltd). The leaf surface temperature was recorded by the same means at intact and damaged parts of sun-exposed and shaded leaves at different times of the day and insolation conditions. Relative air humidity within the canopy was measured with Infrared Psychrometer AZ 8857 (AZ Instruments, Taiwan) 5–10 cm away from the examined leaf blade.

Fresh and dry mass (per unit of leaf area) of intact parts of infested leaflets was also determined for crude assessment of biomass consumption by larvae.

## Results

Traces of leaf-mining by the first-generation larvae appeared in the middle of May 2009 but in early June 2013 (probably due to cold April). Final moulting of the first generation was observed 16–20th of July and early mines of the next-generation larvae at the end of July in both 2009 and 2013. The mines were growing until 13th September 2013 (some of the observed leaves had fallen a week earlier) occupying up to about 25% of the total leaf area (quartile range: 15–31%).

**Fig. 2:**
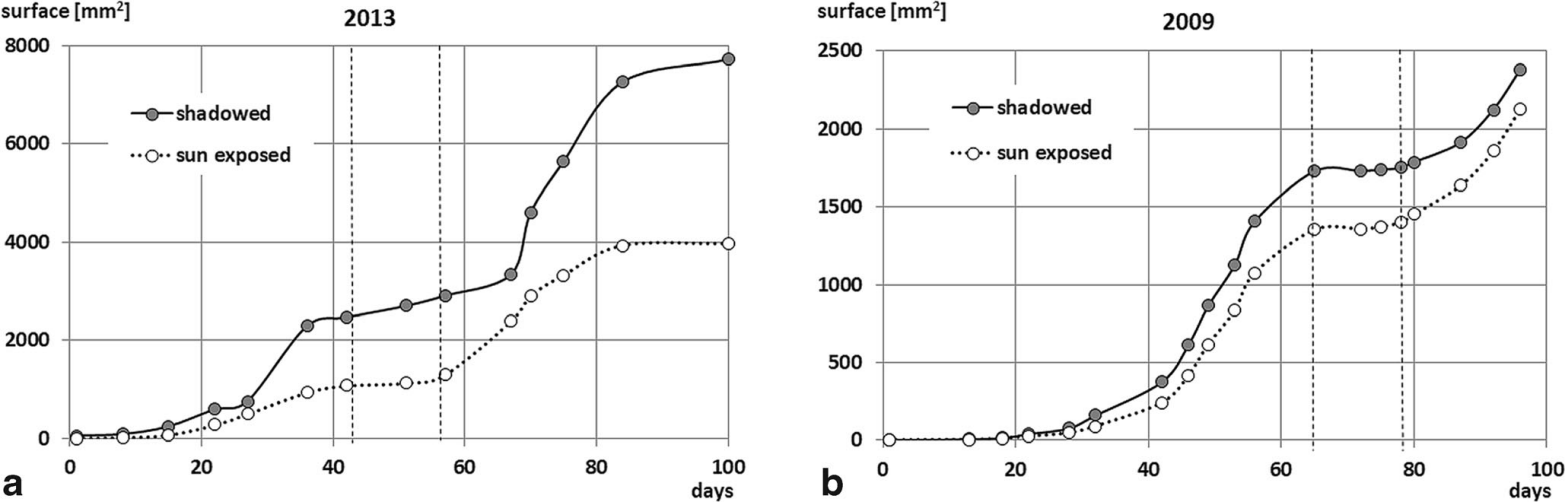
Total mine surface increase on the sun-exposed and shaded leaves of *Aesculus hippocastanum* during the development of two consecutive generations of *Cameraria ohridella* in 2013 (**a** sum of mines area per leaf) and 2009 (**b** mine area of selected mines on the leaf)

Two independent developmental datasets—measurements of mines on particular leaflets (in 2009) and total mined area of selected leaves (in 2013—allowed construction of a reliable mathematical model of *C. ohridella* development on horse-chestnut leaves. The mean mine area’s growth with time followed a double S-shaped curve with ‘plateau phase’ between consecutive generations, which corresponded to pupal stage and adult emergence (Table 1, relative values; Fig. 2, absolute values). Larger area mines occurred on shaded than on the sun-exposed leaves (Fig. 2; Tables 1 and 2).

**Table 1:** Relative mean area of *Cameraria. ohridella* mines (rA) on sun-exposed and shaded leaves of *Aesculus hippocastanum* and the corresponding sum of degree-days during the development of two consecutive generations of the pest in 2013 (changes of the whole mined area of the leaf) and 2009 (changes of selected mines area on particular leaves)

Date		Day	∑ degree-days	Relative values of mine size/area on leaves (rA)			
				Sun exposed		Shaded	
				Mean	SD	Mean	SD
2013							
2013-06-06	I generation	1	15.6	0.003	0.006	0.021	0.024
2013-06-13		8	163.9	0.019	0.014	0.034	0.035
2013-06-20		15	337.6	0.067	0.046	0.097	0.117
2013-06-27		22	482.7	0.257	0.193	0.239	0.166
2013-07-02		27	578.7	0.466	0.520	0.306	0.206
2013-07-11		36	790.7	0.872	0.714	0.927	0.718
2013-07-17		42	909.7	1.000	0.835	1.000	0.735
2013-07-26	II generation	1	25.9	0.049	0.044	0.053	0.060
2013-08-01		7	200.8	0.107	0.063	0.100	0.079
2013-08-11		17	486.0	0.472	0.218	0.197	0.105
2013-08-14		20	547.0	0.644	0.272	0.480	0.306
2013-08-19		25	662.1	0.786	0.267	0.717	0.533
2013-08-28		34	827.6	0.988	0.299	0.864	0.509
2013-09-13		50	1108.4	1.000	0.290	1.000	0.523
2009							
2009-05-20	I generation	1	16.0	0.001	0.000	0.000	0.000
2009-06-01		13	183.5	0.003	0.003	0.003	0.002
2009-06-06		18	239.1	0.009	0.006	0.008	0.005
2009-06-10		22	308.5	0.019	0.014	0.023	0.011
2009-06-16		28	399.8	0.035	0.026	0.044	0.021
2009-06-20		32	462.8	0.066	0.058	0.092	0.044
2009-06-30		42	635.9	0.178	0.098	0.217	0.081
2009-07-04		46	720.6	0.306	0.141	0.353	0.117
2009-07-07		49	780.9	0.454	0.204	0.499	0.156
2009-07-11		53	842.5	0.615	0.279	0.650	0.199
2009-07-14		56	898.8	0.794	0.370	0.813	0.259
2009-07-23		65	1089.1	1.000	0.474	1.000	0.372
2009-07-30	II generation	1	21.0	0.001	0.001	0.000	0.000
2009-08-02		4	83.6	0.005	0.005	0.001	0.001
2009-08-05		7	141.3	0.012	0.012	0.004	0.001
2009-08-07		9	180.2	0.027	0.024	0.009	0.004
2009-08-14		16	300.8	0.077	0.051	0.029	0.012
2009-08-19		21	398.8	0.138	0.079	0.062	0.028
2009-08-23		25	475.0	0.211	0.103	0.101	0.041
2009-08-28		*30*	*708.2*	*…*	*…*	*…*	*…*
2009-09- 01		*34*	*775.2*	*0.995			
2009-09-05		*38*	*839.8*	*…*	*…*	*…*	*…*
2009-09-08		*41*	*889.5*			*0.995	
2009-09-11		*44*	*946.2*	*…*	*…*	*…*	*…*

Relative mine surface area (rA) during the given generation’s growth was calculated against maximal value of average mine surface for the given generation. In 2009, final values of mine size (rA = 0.995) for the second generation (marked by *) were estimated from Verhulst formula (see text for explanation)

**Table 2:** Percentage of total leaf surface mined in *Aesculus hippocastanum* leaves during the experiment

	% of leaf surface mined in 2013				% of leaf surface mined in 2009			
	At the first day		At the last day		At the first day		At the final day (*)	
	Sun	Shadow	Sun	Shadow	Sun	Shadow	Sun	Shadow
Median	0.006	0.084	18.5	26.5	0.075	0.110	13.5	12.0
Q25	0.004	0.061	15.6	22.4	0.065	0.085	10.4	11.1
Q75	0.007	0.165	22.6	30.7	0.335	0.170	16.8	15.8

The values ‘on the last day’ (of observation) represent the sum of damage areas caused by two generations larvae; (*) observations ceased due to accidental damage of the tested foliage; Q25 and Q75, the first and third quartiles

**Fig. 3:**
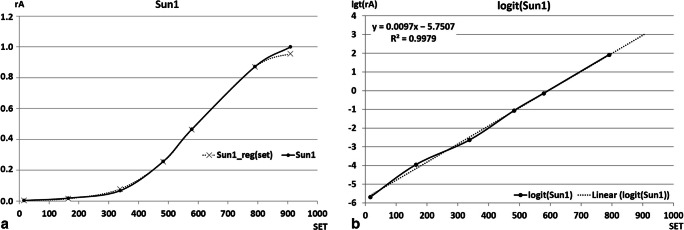
Steps of the computation procedure applied to the mine size of the first generation of *Cameraria ohridella* on sun-exposed leaves in 2013. **a** Mean relative mine area [rA] plotted against sum of effective temperatures [SET] in degree-days, Sun1 (solid line) – rA obtained from measurements, Sun1_reg(set) (dashed line) – rA computed from logit-based linear regression; **b** Logit-transformed mean relative mine area [lgt(rA)] plotted against sum of effective temperatures [set] in degree-days, logit(Sun1) (solid line) – rA experimental data after logit transformation, linear_reg(set) (dashed line) – logit-based linear regression curve optimised with LSD method

The observed ‘plateau’ on the curve provided a reference value for calculating relative mine growth (from the raw data) for the first generation. This step made the results independent of a random selection of the leaflets and suitable for constructing the mathematical model of development (see Fig. 3 as an example of such transformation).

### Modelling of *Cameraria ohridella* development on horse-chestnut leaves

The standardised data for each generation of *C. ohridella* larvae were used to find the Verhulst logistic equation best fitted to the experimental data. Logit transformation followed by regression analysis yielded the parameters of the Verhulst equation. They were similar for both the leaflets and the whole leaves datasets (Tables 3 and 4; Figs. 2 and 3). We used these parameters to fill the missing developmental data of the second-generation larvae in 2009. The set data were 774.5 and 901.3 degree-days for development of the mine to rA = 0.995 for sun-exposed and shaded leaves, respectively. Estimated range of degree-days sum for these calculated values was 751–835 (2009-09-01) in sunny and 837–964 (2009-09-08) in shaded conditions. Such estimation accuracy corresponds to about 4–5 days of larval development (Table 1).

**Table 3:** Parameters of linearised Verhulst equation (Eq. (4)) based on the logistic model of mine area increase dynamics in two generations (1 and 2) of *Cameraria ohridella* larvae dwelling in isolated (Sun) and shaded (Shade) leaves in 2013 and 2009

Group	Days		Degree-days (‘set’)					
	*r*	*a*	*r*	*a*	*R*	*E*	SE	*n*
2013_Sun1	0.214	5.81	0.00969	5.751	0.999	0.0772	0.00022	6
2013_Shade1	0.172	4.60	0.00780	4.558	0.960	2.0478	0.00113	6
2013_Sun2	0.218	3.58	0.00862	5.446	0.966	2.3545	0.00116	6
2013_Shade2	0.151	3.25	0.00602	4.569	0.964	1.1926	0.00082	6
2009_Sun1	0.149	7.44	0.00906	7.083	0.996	0.5613	0.00027	11
2009_Shade1	0.154	7.45	0.00900	6.860	0.993	29.6503	0.00194	11
2009_Sun2	0.207	6.10	0.01115	7.637	0.946	1.2012	0.00119	7
2009_Shade2	0.222	7.34	0.01192	8.983	0.981	0.9399	0.00106	7

*r* slope and *a* intercept of regression, *R* correlation coefficient, *E* and *SE* error and standard error of estimation, *n* number of corresponding observations. Values of equation parameters are calculated for days, and accumulated degree-days (sum of effective temperatures, SET), *K* upper asymptote of equation (maximal value of relative mine surface), and corresponding parameter in Verhulst equation assumed as K = 1

**Table 4:** Probability of differences between pairs of logistic regressions coefficients for mine area (covariance analysis model). Significant differences estimated with Bonferroni adjustment for *k* = 64 and *p'* = 0.000781 (marked in bold)

	2013_Sun1	2013_Shade1	2013_Sun2	2013_Shade2	2009_Sun1	2009_Shade1	2009_Sun2	2009_Shade2
2013_Sun1		0.02456	0.13713	0.00354	**0.00014**	**0.00030**	0.00119	0.00244
2013_Shade1	0.02456		0.14488	0.00883	**0.00022**	**0.00049**	0.01149	0.00254
2013_Sun2	0.13713	0.14488		0.00257	**0.00050**	0.00127	0.07476	0.01305
2013_Shade2	0.00354	0.00883	0.00257		**0.00013**	0.00243	0.00243	0.00327
2009_Sun1	**0.00014**	**0.00022**	**0.00050**	**0.00013**		0.03745	**0.00016**	0.00098
2009_Shade1	**0.00030**	**0.00049**	0.00127	0.00131	0.03745		**0.00043**	0.00275
2009_Sun2	0.00119	0.01149	0.07476	0.00243	**0.00016**	**0.00043**		0.00268
2009_Shade2	0.00244	0.00254	0.01305	0.00327	0.00098	0.00275	0.00268	

### Effect of leaves exposure to sunlight on the dynamics of mine dwelling

During both generations observed in 2013, shaded leaves appeared more severely damaged than sun-exposed ones, but the Kruskal-Wallis test showed no significant difference (Table 2). Estimated time and number of degree-days necessary for the emergence of *C. ohridella* larvae suggested longer development of the second (summer) generation than the first (spring) one (Table 1, Fig. 2). However, regression values did not reveal significant effects of possible causal factors—neither the time of development nor the sum of degree-days (Tables 3 and 4). These results suggest that more data are necessary to resolve whether the inter-generation difference exists.

Fresh and dry mass (per area unit) of the leaf blade was higher for sun-exposed leaves than for shaded ones, suggesting their higher calorific and/or nutritional value. This difference increased with the leaf senescence (Table 5).

**Table 5:** Fresh and dry mass (mean ± standard deviation) of leaf blade (mg·mm^−2^) in sun-exposed and shaded leaves in distinct points of vegetation period. The same letters denote homogeneity of means among fresh mass (a, b) and dry mass (A, B, C) samples

Date	Fresh mass [mg/mm^2^]		Dry mass [mg/mm^2^]		Percent of dry mass	
	Sun-exposed	Shaded	Sun-exposed	Shaded	Sun-exposed	Shaded
2015-07-01	11.8 ± 3.1^a^	9.2 ± 0.2^b^	4.5 ± 1.3^A^	2.3 ± 0.1^B^	38%	19%
2015-08-25	13.2 ± 1.1^a^	9.9 ± 1.7^b^	5.1 ± 0.7^A^	3.0 ± 0.9^B^	39%	23%
2015-09-23	14.0 ± 2.8^a^	8.0 ± 0.5^b^	6.3 ± 1.3^C^	2.8 ± 0.3^B^	45%	20%

### Leaf and mine microclimate

The temperature of sun-exposed leaves was higher than air temperature already in the morning (3 °C at 7 AM) and exceeded 6 °C about noon and early afternoon (3 PM). On the contrary, the temperature of shaded leaves was 1 °C lower than the morning’s air temperature and as much as 4 °C lower by midday. Hence, the difference between sun-exposed and shaded leaves exceeded 11 °C during the midday and was higher than changes in air temperature (Table 6).

**Table 6:** Intact leaf and air temperature [°C] in sun-exposed (sun) and shaded (shadow) places in the canopy of *Aesculus hippocastanum* during sunny midsummer day

		Leaf				Air				Leaf - air difference			
		E	S	W	N	E	S	W	N	E	S	W	N
Shadow	Morning	18.6	20.1	18.1	17.7	18.6	21.0	18.4	18.5	0.0	−0.9	−0.2	−0.8
	Midday		25.0	25.1	24.5		26.6	29.0	27.8		−1.6	−3.9	−3.4
	Evening		22.3	22.9	21.5		24.0	22.9	22.9		−1.6	0.0	−1.4
Sun	Morning	23.1				19.6				3.5			
	Midday		32.8	36.7	32.0		27.8	30.1	28.2		4.9	6.6	3.9
	Evening		21.9	28.9			22.7	26.5			−0.8	2.4	
Sun-shadow difference	Morning	4.5				1.0				3.5			
	Midday		7.8	11.6	7.5		1.2	1.1	0.3		6.5	10.5	7.2
	Evening		−0.4	5.9			−1.3	3.6			0.9	2.4	

Date of measurement: 2015-07-16–2015-07-19; morning = 06:50–07:30; midday = 10:10, 11:00, 14:10, 15:10–15:30; evening = 17:00, 18:00, 19:50–20:20; E, S, W, N—position of the leaf blade within the canopy towards cardinal points of the horizon

**Fig. 4:**
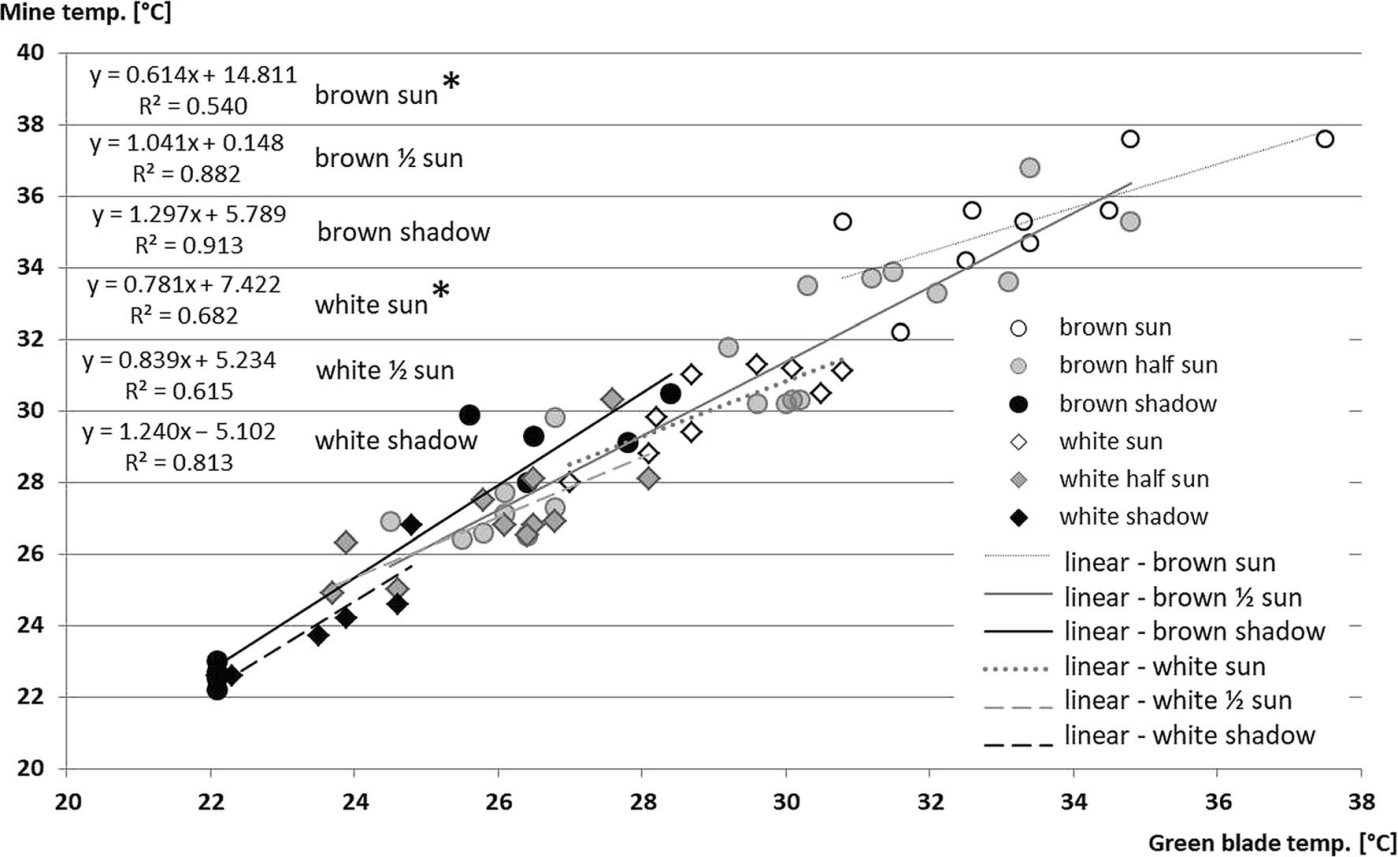
Relationships between the temperature of green intact leaf blade (0X axis) and mine surface (brown, old mine; white, fresh mine) in different light conditions (sun/half-sun/shadow) (for explanation, see M&M). (The asterisk (*) denotes significant difference between marked lines)

Sun rays falling on the intact leaf surface raised its temperature by 5–6 °C within a minute. Damaged leaf area had even greater temperature fluctuations in response to sunlight. Statistical analysis (ANCOVA) revealed a significant effect of mine ‘type’ (white or brown) and insolation on the mine temperature. The surface temperature of new mines was like an intact blade but 2.8 ± 1.7 °C higher in brown, empty mines exposed to sunlight (*p* = 0.009). Mines on shaded leaflets had 0.2 ± 0.1 °C lower surface temperature than undamaged areas (Fig. 4).

Relative humidity within the horse-chestnut canopy ranged from 43 to 69% (lower and upper quartile) and was higher during morning and evening hours and lowered at noon and early afternoon.

These results suggest another unknown factor responsible for a similar rate of the larvae development on shaded and sun-exposed leaves (see ‘Discussion’).

## Discussion

Moths of *C. ohridella* in Balkans emerge at the end of March, and caterpillars of the last, third-generation finish their feeding by mid-November. Pupal and adult stages overlap by 10 to 30 days, while moths and next-generation larvae overlap by 10–20 days. Although 40 days is sufficient for individual larvae to reach pupal stage, the first, second, and third generations need 50, 60, and 90 days, respectively, to complete larval development (Dimic et al. 2000; Samek 2003; Girardoz et al. 2007; D’Costa et al. 2013).

The Ukrainian population of *C. ohridella* (green stands in Kharkiv) also has three generations, and their development depends on the prevailing temperature conditions. Completion of larval stage required on average 22.8, 16.8, and 34.6 days for subsequent generations that corresponded to 203.5, 193.8, and 262.3 degree-days (effective temperatures calculated over 10 °C threshold value), respectively (Meshkova and Mikulina 2013). The detailed study performed by the authors in five consecutive years (2008–2012) revealed that larval developmental time correlated better with the sum of corresponding positive temperatures than the sum of effective temperatures (*r* = 0.882 vs *r* = 0.777 and *r* = 0.944 vs *r* = 0.921 for larvae of the first and second generation, respectively). Moreover, duration of the third-generation larvae did not correlate with positive temperatures but negatively correlated (*r* = −0.891) with effective temperatures instead, during the analysed period, suggesting an involvement of other environmental factors shaping the pest’s development. These results support our assumption of 0 °C as a basis for calculations of *C. ohridella* thermal requirements.

Häffner and Schroer (2011) suggested that the horse-chestnut leaf-miner may develop faster under warmer and artificially illuminated microclimate yielding three to four generations in Berlin and up to five in warmer regions. However, completion of development by subsequent generations may also depend on nutritional value and defensive allomones content of the leaves. Shaded leaves within the canopy contain lower amounts of these secondary metabolites (Yang et al. 2018; Jagiełło et al. 2019). Moreover, larger size mines made by the second-generation larvae, irrespective of the light conditions, suggest deterioration of nutritional quality with leaf age (Table 1; Figs. 2 and 3) (Samek 2003; Girardoz et al. 2007).

The concept of sum of effective temperatures (or similar indices like growing degree-days) as a predictor of development rate of organisms in the ambient environment was criticised due to large circadian variability of thermal conditions and insolation (Solantie 2004). Nevertheless, many authors used this concept for numerous species, particularly important in agriculture (Hanula et al. 1987; Zou et al. 2004; Bergant and Trdan 2006; Doganlar 2008; Murray 2008; Dixon et al. 2009; Jarosík et al. 2011). Complex interactions among ambient thermal conditions and developmental rate in insects within the range of natural environmental temperature variability (10–35 °C) can be simplified by the linear day-degree sum model (‘law of total effective temperatures’) (Damos and Savopoulou-Soultani 2012) or mean daily temperature (Nielsen et al. 2016). In the presented work, sum of degree-days was estimated according to almost ‘historical method’—calculation of the mean daily temperature from eight measurements taken every 3 h at the nearest weather station (Pruess 1983; Felber et al. 2018). Despite some disadvantage (lack of local radiation and microclimate variability data on experimental stands), the method gives a good approximation of conditions determining the development of mine-dwelling larvae, confirmed by high values of correlation coefficients for presented equations. Hence, our developed model should confirm its predictive potential also when applied to seminatural conditions like greenhouse experiments (see Jagiełło et al. 2019).

Some studies suggest that leaf-miners and leaf-gallers prefer sun-exposed leaves over those growing in the shade (Dai et al. 2013; Alexandre et al. 2018), but Horváth and Benedek (2009) pointed out that the preference of *C. ohridella* for shaded vs sun-exposed leaves likely results from seasonal variation of leaf microclimate and/or nutritional value of the leaf tissue. The role of microclimate variability on insect development was analysed by Rebaudo et al. (2016), who concluded ‘…that the model implemented with microclimatic data best predicted observed pest abundances for [their] study sites, but was less accurate than the global dataset model when performed at larger scales’. Therefore, the mismatch between organisms’ size and the scale at which microclimate data are collected should be considered.

In spring, moths of *C. ohridella* lay eggs preferably on the leaves located in lower and middle parts of the canopy. Although larger leaves may offer habitat for more larvae, small leaves yielded a greater number of hatched moths per unit of leaf weight (Horváth and Benedek 2009). Multiplying mines area (Table 1) by blade biomass (per unit area) resulted in similar biomass consumed by larvae irrespective of the leaves insolation (Table 2). Similar results were obtained in glasshouse experiment on infested horse-chestnut saplings kept in ‘high’ and ‘low’ light conditions (Jagiełło et al. 2019). Much larger biomass consumed by the second generation’s larvae, revealed in our study, suggests decreased nutritional quality of the leaf tissues. This age-dependent change might have included either depletion of primary metabolites or increased amounts of secondary compounds.

Frequency of radiation reflected by plant leaves (8970–9890 nm) is shifted towards longer wavelengths in respect to solar radiation (300–2000 nm) reaching their surface (Peñuelas and Filella 1998; IRT_protocol 2015). Thus, sun-exposed leaves’ temperature could rapidly rise by as much as 10 °C relative to shaded leaves (Ansari and Loomis 1959; Lomas et al. 1971; SEOS 2015). Our data similarly indicated that the sun-exposed mines’ temperature exceeded air temperature by 10 °C (even 20 °C in a single measurement) within minutes.

Leaf-dwelling larvae encounter specific mine environments that may differ from macro- and microclimatic conditions over the leaf surface, due to chilling effect of leaf transpiration and local fluctuations of insolation level within the foliage. When feeding on mesophyll, the mining larva removes the tissue responsible for absorption and reflectance of 40–90% of radiation energy (Billings and Morris 1951; Jones and Rotenberg 2001; Slaton et al. 2001; Merzlyak et al. 2002). Remaining a semitransparent window increases the visible and infrared sun rays’ penetration into the mine chamber that rises its temperature by several degrees over adjacent tissue temperature that we observed in our study. Moreover, the growth of mine chamber size along with larva development may further disturb this microenvironment equilibrium. It may involve elevated humidity and concentration of carbon dioxide (Pincebourde and Casas 2006a, b) and/or smaller and delayed heat loss due to reduced gas and water exchange in both intact and mined parts of infested leaves (Raimondo et al. 2005). In such conditions, metabolic rate of sun-exposed larvae, assessed on the CO_2_ release, may be up to five times higher than in non-exposed ones (Pincebourde and Casas 2006a, b). Thermal conditions within the mine may also be influenced by the colour change of remaining leaf tissue, caused by frass and cohabiting fungal and bacterial leaf parasites (Akimov et al. 2003).

Elevated temperatures in the mine may accelerate larval development (Pincebourde and Casas 2006b). Still, they may also increase the risk of their overheating or necessity to conform to unfavourably high temperatures within the mine. Such conditions prevail in large and oldest mines (Akimov et al. 2003) inhabited by older instars considered more resistant (Pincebourde and Casas 2015). Additionally, the mine environment also displays greater diurnal variability in microclimate than intact leaf tissue that, paradoxically, may significantly accelerate ectotherm development, as was demonstrated by Niehaus et al. (2012), hence further differentiate larval growth rate in the insolated and shaded mines.

Despite that, insects’ vital functions under variable microclimatic conditions conform to Shelford’s law of ecological tolerance, characterised by a right-skewed normal distribution (Dixon et al. 2009). As Pincebourde and Woods (2012) pointed out, ‘leaf microclimate can provide suitable microhabitats in an unfavourable climate, and conversely, they can bring a species to local extinction in what would seem to be an otherwise favourable climate’.

Our results showed that either local microclimatic conditions within the mine or higher nutritive values of sun-exposed leaf tissues affected the rate of *C. ohridella* larvae development and moulting. Nevertheless, as demonstrated by our model, the time or crude sum of degree-days necessary to complete larval stage did not differ substantially between the insects inhabiting sun-exposed and shaded leaves. Thus, the model can serve as a good predictor for the timing of pest management actions.

The Verhulst logistic equation appears to be a useful model for describing numerous biological processes, especially population growth with limited resources (Tsoularis 2001; Tsoularis and Wallace 2002). Recently, Joshi et al. (2016) applied similar formulas to compare developmental time among three generations of codling moth (*Cydia pomonella* L.) in cultivated and abandoned apple orchards. Here, we have shown that it can be successfully applied to model development of consecutive generations of leaf-mining larvae as well.

## Conclusions

Our study suggests that leaf-miner larvae can effectively compensate for microhabitat variability during their life cycle. Significant differences in microclimatic conditions and nutritional quality were ‘resolved’ by enhanced feeding on shaded leaves that resulted in a similar duration of larval development in both shaded and sun-exposed leaves. An elaborated mathematical model offers a convenient tool to explain whether similar compensatory reactions occur during larval development in other leaf-mining insects in variable natural conditions.
