# Correction to: Towards understanding *Cameraria ohridella* (Lepidoptera: Gracillariidae) development: effects of microhabitat variability in naturally growing horse-chestnut tree canopy

**DOI:** 10.1007/s00484-024-02656-y

**Published:** 2024-03-15

**Authors:** Piotr Łaszczyca, Mirosław Nakonieczny, Andrzej Kędziorski, Agnieszka Babczyńska, Marta Wiesner

**Affiliations:** 1 Faculty of Natural Sciences, Institute of Biology, Biotechnology and Environmental Protection, Animal Physiology and Ecotoxicology, Team, University of Silesia in Katowice, PL, Bankowa 9, Katowice, 40-007 Poland; 2 Główny Instytut Górnictwa (GIG), plac Gwarków, 1, Katowice, 40-166 Poland

The article was published with an error in Eq. 3. Where the equation appears as:

Logit linear transformation was based on the following formulas (Eqs. 3 and 4) (McDonald 2014):

3 $$Logit\,({N_t}) = \frac{{In{N_t}}}{{1 - {N_t}}}$$

The equation should instead appear as:

Logit linear transformation was based on the following formulas (Eqs. 3 and 4) (McDonald 2014):

3 $$Logit\,({N_t}) = In\frac{{{N_t}}}{{1 - {N_t}}}$$

This correction stands to correct the original article. The original article has been corrected.

